# From hardware store to hospital: a COVID-19-inspired, cost-effective, open-source, in vivo-validated ventilator for use in resource-scarce regions

**DOI:** 10.1007/s42242-021-00164-1

**Published:** 2021-09-22

**Authors:** Matthew H. Park, Yuanjia Zhu, Hanjay Wang, Nicholas A. Tran, Jinsuh Jung, Michael J. Paulsen, Annabel M. Imbrie-Moore, Samuel Baker, Robert Wilkerson, Mateo Marin-Cuartas, Danielle M. Mullis, Y. Joseph Woo

**Affiliations:** 1 Department of Cardiothoracic Surgery, Stanford University, Falk Cardiovascular Research Building CV-235, 300 Pasteur Drive, Stanford, CA 94305-5407 USA; 2 Department of Mechanical Engineering, Stanford University, Stanford, CA USA; 3 Department of Bioengineering, Stanford University, Stanford, CA USA; 4 Department of Comparative Medicine, Stanford University, Stanford, CA USA; 5 University Department of Cardiac Surgery, Leipzig Heart Center, Leipzig, Germany

**Keywords:** COVID-19, Ventilator, Open-source

## Abstract

Resource-scarce regions with serious COVID-19 outbreaks do not have enough ventilators to support critically ill patients, and these shortages are especially devastating in developing countries. To help alleviate this strain, we have designed and tested the accessible low-barrier in vivo-validated economical ventilator (ALIVE Vent), a COVID-19-inspired, cost-effective, open-source, in vivo-validated solution made from commercially available components. The ALIVE Vent operates using compressed oxygen and air to drive inspiration, while two solenoid valves ensure one-way flow and precise cycle timing. The device was functionally tested and profiled using a variable resistance and compliance artificial lung and validated in anesthetized large animals. Our functional test results revealed its effective operation under a wide variety of ventilation conditions defined by the American Association of Respiratory Care guidelines for ventilator stockpiling. The large animal test showed that our ventilator performed similarly if not better than a standard ventilator in maintaining optimal ventilation status. The FiO_2_, respiratory rate, inspiratory to expiratory time ratio, positive-end expiratory pressure, and peak inspiratory pressure were successfully maintained within normal, clinically validated ranges, and the animals were recovered without any complications. In regions with limited access to ventilators, the ALIVE Vent can help alleviate shortages, and we have ensured that all used materials are publicly available. While this pandemic has elucidated enormous global inequalities in healthcare, innovative, cost-effective solutions aimed at reducing socio-economic barriers, such as the ALIVE Vent, can help enable access to prompt healthcare and life saving technology on a global scale and beyond COVID-19.

**Supplementary Information:**

The online version contains supplementary material available at 10.1007/s42242-021-00164-1.

## Introduction

As the world begins to grasp the implications of coronavirus disease 2019 (COVID-19), which often results in the need for mechanical ventilation [1, 2], evidence continues to unfold regarding that mortality rates are disproportionately higher for both racial and ethnic minorities and for those who are economically disadvantaged, and these higher rates are largely implicated with limited access to healthcare [3–6]. As of August 2021, there have been more than four million deaths worldwide attributed to the virus, and many minority groups are grossly overrepresented in these mortalities [4, 7]. Moreover, evolved strains like the Delta variant continue to spread rapidly throughout regions such as India, Great Britain, and the United States, causing new waves of infections and pressures on healthcare systems despite rising vaccination rates. This pandemic highlights the dire need for the international scientific community to address the severe global inequalities that plague low-income nations. Developing creative solutions to improve access to healthcare is a step toward eliminating these disparities, where one area of immediate concern is the shortage of ventilators. In resource-scarce regions with severe outbreaks of COVID-19, there may not be enough ventilators to support critically ill patients [8, 9]. We have seen these shortages greatly impact nations such as India, which has recently experienced a massive surge in COVID-19 cases with nearly 400,000 new cases daily (as of May 2021), and estimates are that only 3–4% of all cases are being detected [10]. The lack of ventilation resources is anticipated to be particularly devastating in regions such as Africa, where 10 countries have zero ventilators, and another 14 countries have 20 or fewer such devices, as there are fewer than 2000 ventilators working to support populations of hundreds of millions, in contrast to the approximately 200,000 ventilators in the United States [11, 12].

In response, we have developed the accessible low-barrier in vivo-validated economical ventilator (ALIVE Vent), which is a cost-effective, rapidly scalable, in vivo-validated, clinically focused and multi-feature solution. Our device can be quickly assembled from standard, commercially available parts and has been validated in anesthetized large animals with efficient operation and no observed detrimental effects. To the best of our knowledge, the ALIVE Vent is the only COVID-19-inspired, non-artificial manual breathing unit (AMBU) bag ventilator to date, which is capable of meeting the American Association of Respiratory Care (AARC) guidelines for mass casualty respiratory failure ventilator stockpiling and has been tested on live large animals. It is electronically driven by compressed air and oxygen and uses our openly available software application to implement precise, reliable control of the most clinically relevant ventilation variables, unlike many AMBU bag solutions [13–15]. The incentive to design the ALIVE Vent is entirely humanitarian and does not pursue any commercial interest. All design materials are made publicly available for free and open use.

**Fig. 1:**
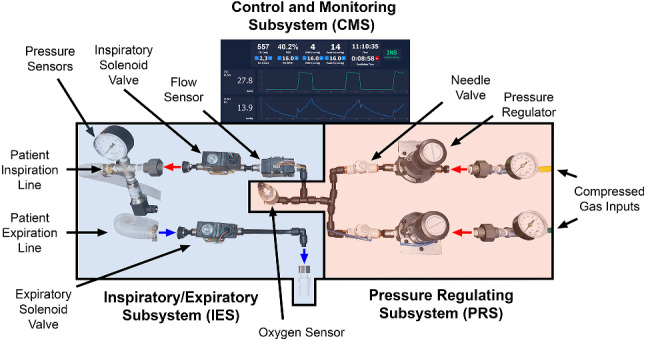
ALIVE Vent with its subsystems and components labeled

**Fig. 2:**
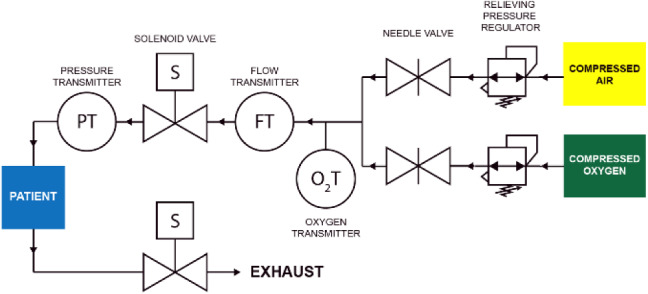
Labeled circuit diagram of the ALIVE Vent. The system is driven via titratable compressed air and oxygen, and the sensors transmit information to a computer for monitoring and control

Our novel device operates using compressed oxygen and air to drive inspiration, while two solenoid valves ensure one-way flow and precise cycle timing. The ALIVE Vent is comprised of three subsystems: (1) the pressure regulating subsystem (PRS), (2) the inspiratory/expiratory subsystem (IES), and (3) the control and monitoring subsystem (CMS) (Fig. 1). Air flow can be visualized via a circuit diagram (Fig. 2). Relieving pressure regulators in the PRS safely reduce the compressed gas pressure, while needle valves accurately titrate the oxygen composition of the gas. Three key sensors: a flow, an oxygen, and a pressure sensor are used to monitor ventilation variables and deliver precise doses of the low-pressure gas via sensor-mediated threshold gating of the solenoid valves. The expiratory port leads to a wall exhaust and a high-efficiency particulate air (HEPA) filtering system, preventing the exhaust of airborne viruses. The CMS consolidates and manipulates all of the components using a computer running our Python software application and a simple circuit board. These sensors and regulators allow users to both monitor and control peak inspiratory pressure (PIP), positive-end expiratory pressure (PEEP), tidal volume (*V*_T_), fraction of inspired oxygen (FiO_2_), respiratory rate (RR), and inspiration to expiration time ratio (I/E).

## Methods

### Design of the ALIVE Vent

The novel hardware structure of ALIVE Vent aims to minimize the number of parts while providing the baseline control and performance capabilities dictated by emergency clinical ventilator guidelines. While most of the parts were sourced from McMaster-Carr Supply Company (Elmhurst, IL, USA), an American industrial supplier, everything was conceived, designed, and assembled from scratch within our laboratory using standard hardware store tooling.

As mentioned above, our original ventilator design includes three subsystems: (1) the PRS, (2) the IES, and (3) the CMS (Fig. 1)*.* Functionally, the PRS receives the input of compressed gas and translates it to the appropriate pressure and oxygen titration level for the patient. This subsystem is comprised of four main components: (1) gas line connectors, (2) pressure regulators, (3) area restriction flow regulators, and (4) oxygen sensor. Since our test was performed using high-pressure air and oxygen lines (approximately 50 psi), our gas connectors use Ohmeda valves and stainless-steel union fittings that connect to the pressure regulators through in-line mechanical pressure gauges. The two relieving mechanical pressure regulators were specifically selected as they precisely convert a wide range of compressed gas pressures to low pressures (0–2 psi). These pressure regulators were attached to flow regulators (in the form of needle valves) via push-to-connect fittings and firm polyurethane tubing for an additional level of control to independently titrate the FiO_2_, which is otherwise not required for full titration functionality. The needle valves operate by restricting the area of the tube that controls the volumetric flow rate of gas. Finally, these two lines were connected to a zirconia oxygen sensor. While medical grade, disposable, electro-galvanic oxygen sensors specifically made for ventilators can and should be used, we did not want to include such a part in our experimental application, which may be in limited supply. Ultimately, the final output of the PRS was a stable, regulated, low-pressure, oxygen-titrated gas supply that is specifically tuned to the patient’s requirements.

Upon receiving the PRS input of modulated gas supply, the IES functions to deliver precise doses of inhaled gas to the patient in a unidirectional configuration and is equipped with flow monitoring capabilities. The IES is comprised of two electrically controlled solenoid valves, an inspiratory flow sensor, a pressure sensor and gauge, and hose barbs for interfacing with split medical ventilation tubing delivered to the patient. The use of two solenoids and split tubing ensures unidirectional flow through the system, allowing for adequate expulsion of exhaled carbon dioxide. The electronic pressure sensor was connected to the inspiratory solenoid for the continuous monitoring of lung pressure, as it is important to make sure that the pressure does not exceed clinically validated values. In addition, the electronic pressure sensor is critical for monitoring and control of PEEP. The inspiratory flow sensor (Honeywell, Charlotte, NC, USA) was used to identify flow within a range of 0–100 standard liter per minute (SLPM) and calculate the *V*_T_, while an identical, optional expiratory flow sensor can be included for monitoring capabilities. Finally, in our case, the expiratory solenoid valve was connected to a hose barb and medical tubing, which had an output to a negative pressure wall exhaust port in our operating room. If this is not feasible in an emergency setting, HEPA filters should be used at the exhaust port to prevent the exhaust of any airborne virus.

The CMS actively controls and monitors the components of the other subsystems, and it is comprised of a computer running our custom Python software application, a prototyped circuit board, and a power supply. The inputs to the CMS include the oxygen sensor from the PRS and the flow and pressure sensors from the IES, while the outputs of the CMS are the actuations of the two solenoid valves in the IES. The inputs and outputs are consolidated on our circuit board, and each of them are processed via the on-board microcontroller. The circuit board contains two N-channel MOSFETs connected to the microcontroller and a 24 V power supply, which are used to actuate the solenoids. The sensor information is measured via either the Inter-Integrated Circuit (*I*^2^*C*) communication protocol or an analog-to-digital converter. A 12 V linear voltage regulator powers the 12 V pressure transducer through a 24 V source. The microcontrollers serially communicate with our software platform with a sampling rate of approximately 200 Hz, and the data are displayed and monitored on our graphical user interface designed using PyQt5. We advise using a sampling rate > 100 Hz, as lung conditions can change rapidly in a ventilation environment, and the root mean square error for pressure control is directly related to the sampling rate. The software application applies additional feedback control to the solenoid valves based on these metrics. For example, PEEP and PIP are controlled via sensor-mediated threshold gating of the solenoid valves based on the lung pressure readings resulting in pressure control, which is also the way we chose to operate our ventilator. Moreover, using the flow sensors as input, *V*_T_-gating allows for volume control, which can be managed by the software platform. While this control system has been designed in a prototyping capacity, further development should address the creation of additional alarming and safety protocols in both the software and the electronics for better patient safety and easier application by the physician.

### ALIVE Vent functional testing

In order to characterize the functional capabilities of the ALIVE Vent and contextualize these capabilities into clinically relevant standards, we first identified the key operating features provided by the AARC SARS CoV-2 guidance document (https://www.aarc.org/wp-content/uploads/2020/03/guidance-document-SARS-COVID19.pdf). This document outlines the “standard functional performance requirements for ventilators stockpiled for use in mass casualty respiratory failure,” which have been reproduced in Table S1. To identify whether the ALIVE Vent was capable of fulfilling these standards and to profile its performance across a wide range of ventilation conditions, we used the SmartLung 1000 1L (IMT Analytics, Buchs, Switzerland), an artificial test lung that is capable of providing four levels of adjustable resistance and compliance at a maximum capacity of 1 L. From the four independent SmartLung levels of resistance (5, 20, 50, and 200 mbar/(L·s)) and compliance (10, 15, 20, and 30 mL/mbar at 400 mL *V*_T_), the minimum and maximum *V*_T_ set by the guidance document (50, 750 mL), and three RR levels defined by the document (6, 20, and 35 bpm), we measured the maximum and average inspiratory flows for each of the 96 testing conditions (Table S2).

We did not independently specify the PEEP or FiO_2_ because the ALIVE Vent can produce any range of these two metrics by the nature of its design. More specifically, PEEP is controlled by pressure-gated thresholding of the solenoids, meaning that it can be specified to have any pressure that is less than the final inspiratory pressure. Moreover, FiO_2_ is titrated via direct 50 psi standard compressed air and compressed oxygen connections. These high-pressure lines allow each connection to drive ventilation independently, which means that our ventilator can provide any combination of FiO_2_ values from pure air to pure oxygen, or 21–100%.

It is essential to understand the theoretical operating principle of controlling this device in the clinic. Specifically, our device has been designed to manipulate clinical variables according to the Hagen–Poiseuille equation derived from the Navier–Stokes equations, which describes the flow pressure drop in a long cylindrical pipe under laminar flow conditions (Eq. (1)). This equation dictates that the volumetric flow rate (*Q*) is dependent on the pressure gradient (Δ*p*) and cross-sectional area (*A*) of the tubing, which are manipulated in the ALIVE Vent via the pressure regulators and needle valves in the PRS. Thus, by titrating these components in combination with the *I/E*, one can define the volumetric flow rate and thus the *V*_T_ accordingly. It is important to mention this key operating principle of the ALIVE Vent, because as the lung expands, the pressure gradient drops, resulting in a subsequent reduction in the inspiratory flow. While the effect of this phenomenon can be minimized by increasing the pressure gradient, doing so has downstream effects on *Q*, *I/E*, and PIP.

1 $$Q= \frac{\Delta p{A}^{2}}{8\uppi \mu L},$$

where *µ* is the dynamic fluid viscosity and *L* represents the pipe length.

### Animal care and biosafety

Two male Dorset sheep (sheep 1 weighing 40 kg and sheep 2 weighing 60 kg) were obtained from Joe Pozzi Livestock (Sonoma, CA, USA). Food and water were provided ad libitum until one day prior to the experiment. The animals were handled in accordance with the Guide for the Care and Use of Laboratory Animals published by the US National Institutes of Health (Publication No. 85-23, revised 1996). The experimental protocol (#33819) was approved by the Institutional Animal Care and Use Committee of Stanford University, which is accredited by the Association for the Assessment and Accreditation of Laboratory Animal Care.

### In vivo testing and data collection

The animals were sedated with telazol (Zoetis, Parsippany-Troy Hills, NJ, USA). An oxygen pulse oximeter was applied for monitoring continuous O_2_ saturation and heart rate. Glycopyrrolate (Piramal Critical Care, Bethlehem, PA, USA) was given intramuscularly to reduce salivation. Intravenous access was established through the external jugular vein, and propofol (Hispira, Lake Forest, IL, USA) was administered intravenously to complete the induction. The sheep were placed in ventral recumbency and intubated with a 7-0 endotracheal tube via direct visualization and passage of the endotracheal tube through the vocal cords. Anesthesia was maintained with 1–3% isoflurane (Fluriso, VetOne, Boise, ID, USA) using the commercial ventilator, Moduflex™ Elite Veterinary Anesthesia Machine (Dispomed, QC, Canada), on 100% FiO_2_. Arterial access was obtained via the auricular artery. Twenty minutes prior to ventilator testing, isoflurane was stopped, and propofol was administered to maintain anesthesia. After baseline chest X-rays were obtained, the sheep were connected to the ALIVE Vent. PEEP and FiO_2_ were, respectively, maintained at 2 mmHg and 40%. The pressure, flow, RR, and *I/E* were adjusted to optimize O_2_ saturation and end tidal CO_2_. After 60 min of testing using the ALIVE Vent, sheep were switched to the standard commercial ventilator. Post-experimental chest X-rays were acquired, and the animals were kept on the standard commercial ventilator for another 60 min before being subsequently recovered. Throughout the experiment, arterial blood gas and vital signs were measured for both sheep at baseline and at approximately the 20, 40, and 60-min time points for both the ALIVE Vent and standard ventilator use (Table S3).

## Results

**Fig. 3:**
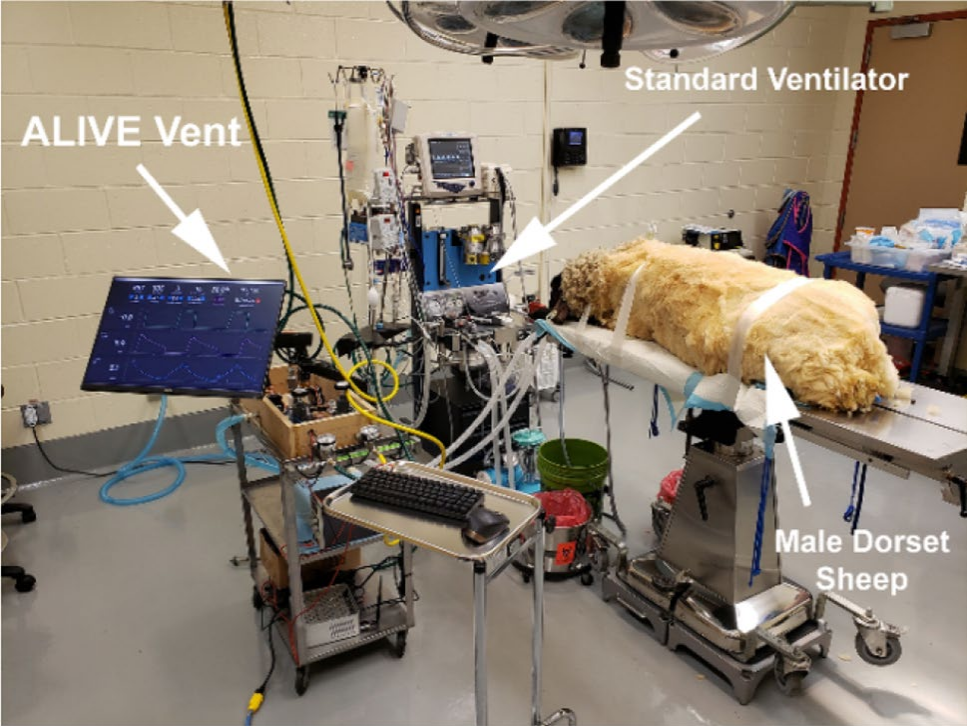
In vivo testing setup including the ALIVE Vent, a standard ventilator, and anesthetized male Dorset sheep

**Fig. 4:**
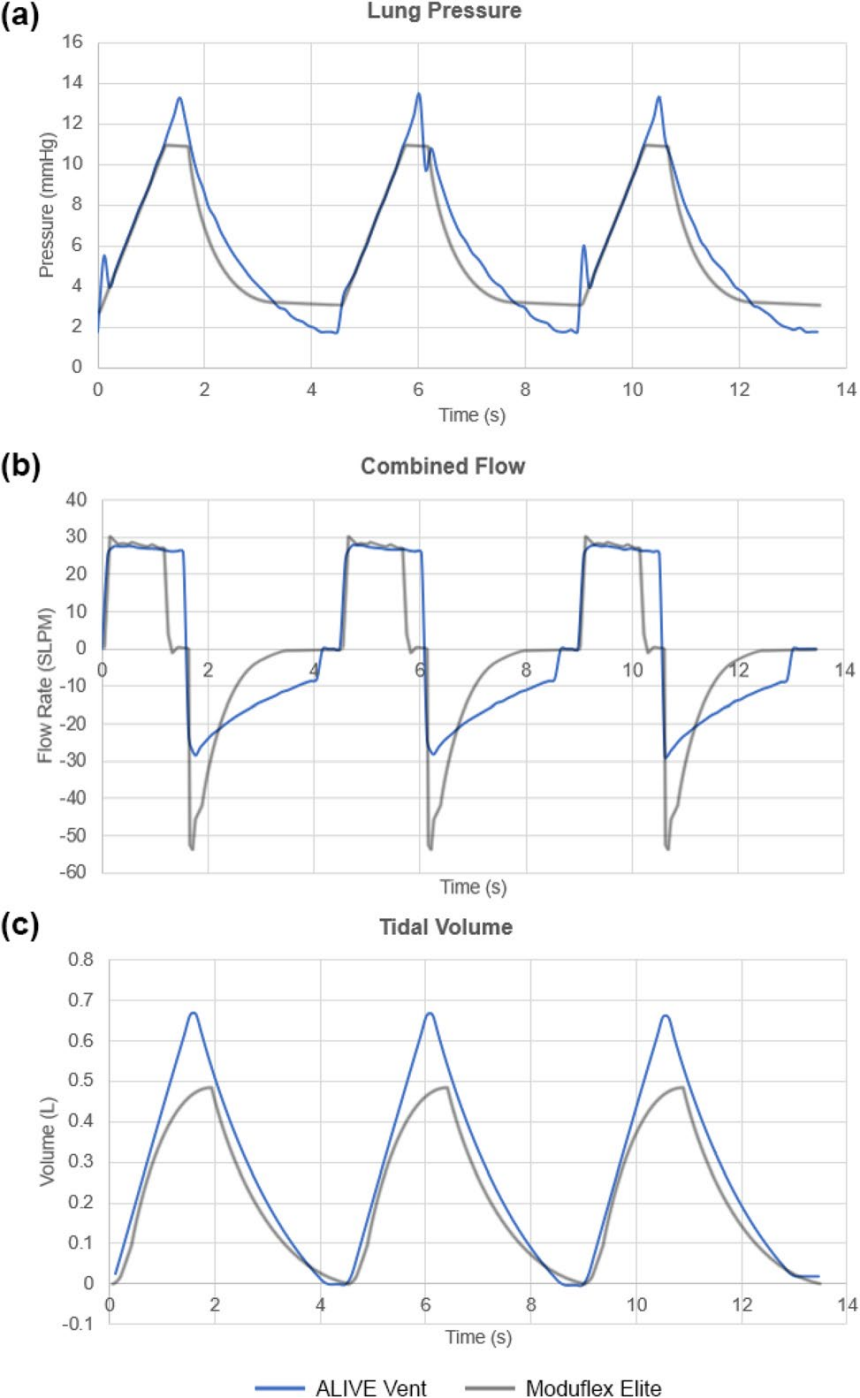
Representative lung pressure (**a**), flow (**b**), and tidal volume (**c**) data recorded for three breathing cycles by the ALIVE Vent and the Moduflex Elite Ventilator. Data were recorded across multiple ventilation conditions, and representative curves in one subplot may not correspond to consistent recording windows

Based on the functional test using the SmartLung 1000 1 L (IMT Analytics, Buchs, Switzerland), we found that the ALIVE Vent was capable of providing all standard performance metrics, set by the AARC COVID-19 Guidance Document, at all lung ventilation conditions specified by the SmartLung (Tables S1 and S2). To validate the proper operation of the ALIVE Vent and to compare it to a standard, commercially available veterinary ventilator, we performed non-terminal, in vivo ventilation testing in two anesthetized male Dorset sheep with a weight difference of 20 kg (Fig. 3). We found that the ALIVE Vent was able to maintain and manipulate the respiratory status by modulating ventilation variables, including RR, *I/E*, PEEP, and PIP. Throughout the animal testing procedure, the ALIVE Vent parameters were successfully maintained within the ranges of 35–45% for FiO_2_, 10–15 bpm for RR, 1.5–3.5 for *I/E*, 1.5–2.5 mmHg for PEEP, and 10–18 mmHg for PIP. Representative lung pressure, flow, and tidal volume data were recorded and graphed for both the ALIVE Vent and the commercial ventilator (Fig. 4).

**Fig. 5:**
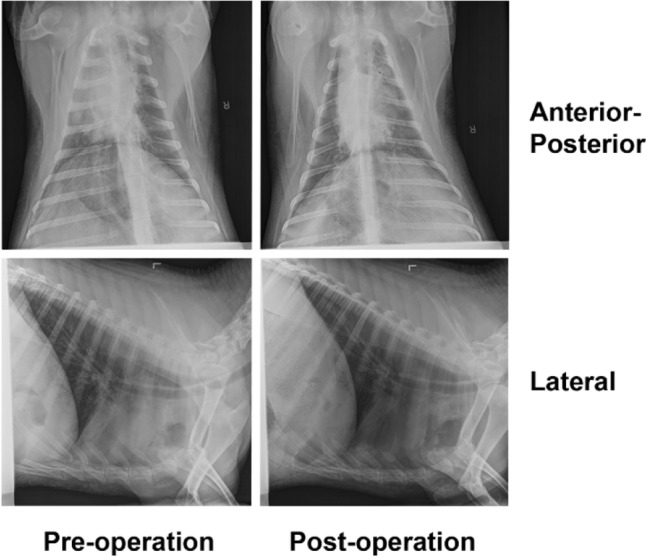
X-ray images of an anesthetized male Dorset sheep pre- and post-operation of the ALIVE Vent

At approximately 40% FiO_2_, the ALIVE Vent maintained the blood partial pressure of oxygen (pO_2_), partial pressure of carbon dioxide (pCO_2_), and pH across all sampled time points at 139.67 ± 6.68 mmHg, 38.77 ± 1.26 mmHg, and 7.43 ± 0.01, respectively. At 100% FiO_2_, the standard ventilator maintained the same metrics at 364.03 ± 56.25 mmHg, 37.78 ± 1.70 mmHg, and 7.45 ± 0.004, respectively, (reported as mean ± standard deviation). The difference in pO_2_ occurred because the standard ventilator did not have oxygen titration capabilities, leading to a set FiO_2_ of 100%. These data proved that the ALIVE Vent performed similarly to if not better than the standard ventilator in maintaining pO_2_, pCO_2_, and pH and that the additional oxygen titration abilities of the ALIVE Vent reduced the hyperoxia experienced by the animal under the standard ventilator (Table S3). Neither pneumothorax and atelectasis, nor other signs of baro- and volutrauma were identified in the post-ventilation X-rays, and vitals were within normal limits throughout the duration of the procedures (Fig. 5). The sheep recovered without any complications.

## Discussion

In this study, we demonstrated the design and in vivo validation of an innovative, cost-effective, stand-alone ventilator that uses commercially available parts. Through SmartLung and large animal testing, we showed that the ALIVE Vent can not only fulfill all ventilation standards set by AARC COVID-19 guidance, but also precisely manipulate and monitor key ventilation metrics, such as FiO_2_, RR, *I/E*, PEEP and PIP, to safely maintain blood gas composition at clinically directed values. Our design and software application are made openly available for rapid and scaled humanitarian implementation, and the technical details can be found in a public online repository (see Data Availability).

Commercial ventilators with similar features to the proposed ALIVE Vent can cost between $25,000 and $59,000 [16]. While pricing will vary based on region and availability, the final cost of parts for our device was approximately $1400, with most components sourced from an American industrial supplier (Fig. S2a). However, this cost can be further reduced to approximately $975 with only slight modifications (Fig. S2b). Many of these components can be commonly found in hardware or plumbing stores and are autoclavable.

When comparing the flow, pressure, and tidal volume data of the ALIVE Vent to a commercially available ventilator, a remarkable similarity is observed in the flow dynamics with a noticeable plateau in the inspiratory flow curve, indicating a resistance to changes in the pressure gradient (Fig. 4b). While there may be small differences in flow gating logic that are dependent on the control algorithm, the striking similarity shows the capability of the ALIVE Vent to replicate the performance of commercial systems. These data and the performance comparison are further corroborated when studying the representative flows of clinical data and guidance documents for ventilation management [17]. Moreover, like other commercial ventilators, the ALIVE Vent contains internal gas blending features to independently titrate the FiO_2_ of the inhaled gas, which is a rare yet essential feature to have in a low-cost emergency ventilator. Overall, the ALIVE Vent is cost-effective, precise, quick to assemble, massively scalable, and only requires access to compressed gases and electricity, which can be provided in distant rural settings using generators, compressors, and gas tanks.

Many COVID-19-inspired ventilator solutions have either used AMBU bags with motorized compression [13–15, 18, 19] or implemented innovative ventilator multiplexing designs [20, 21]. Additionally, recent work has presented the design and development of cheaper, creative solutions, such as the water column PEEP management built with polyvinyl chloride tubing [22]. However, while these systems can potentially serve as emergency devices in the short-term, which provide more effective alternatives than mechanical ventilators and have been described as last resort solutions, they are not ideal as long-term, clinically translatable solutions due to their lack of thorough mechanical profiling and animal testing results. Besides, none of them have been shown to match the rigorous AARC guidelines, and few if any have been tested on large animals or operated in a clinical setting. Moreover, since the ALIVE Vent uses compressed gas and computer-monitored control, it can overcome the many limitations of these alternatives. Specifically, motorized AMBU bags offer rudimentary, imprecise control of ventilation parameters with no ability to titrate FiO_2_. While having lower cost, these systems require the calibrated interpolation of key metrics, such as lung pressure and tidal volume, as opposed to direct transduction, which results in high variability and imprecision in monitoring and controlling key variables that would need to be accurately set to avoid catastrophic lung injury or other failures. Conversely, the ALIVE Vent offers a direct transduction of lung pressure, inspiratory flow, and oxygen levels, allowing for the precise monitoring and control of critical parameters.

Additionally, while ventilator multiplexing has shown in vivo efficacy, the number of parameters that can be independently controlled in such processes is limited [20, 21]. As these solutions operate by individually modulating flow delivered to unique branches from a set ventilator supply, RR, I/E, FiO_2_ and PEEP cannot be separately adjusted for each patient. Furthermore, multiplexers are physically restricted to six branches due to the maximum ventilation volume of most ICU ventilators [21], and they require hospitals to have available ventilators, which is a burden for many developing countries. These are significant confounding factors preventing full ventilation control and the widespread adoption of such systems. On the other hand, the ALIVE Vent is a stand-alone system meant for individual patient use, allowing for maximum, independently tuned control over each patient’s clinically determined unique ventilation requirements.

The main limitation of our study is that the proposed device was specifically designed in a prototyping capacity for our large animal testing application. For example, the compressed gas and exhaust connections were sourced to interface with the wall connections in our operating room facilities, and these conditions may considerably vary between points-of-care. While our lightweight design focuses on reliably implementing core ventilation features, future work must incorporate either adaptive modularity for widespread integration or region-specific implementation suited for a variety of different hospital conditions. A further key limitation is the requirement for access to electricity and pressurized air/oxygen. While many clinical environments are equipped with these resources, in the case when these are unavailable, rudimentary, or even manual, electric generators and lightly compressed air/oxygen cylinders can still provide sufficient voltage and pressure gradients for basic operation because the operational power and pressure requirements for our device are low. Specifically, for the ALIVE Vent, the maximum PIP requirement is on the order of magnitude of 10–100 mmHg, while a standard compressed oxygen cylinder has a pressure of at least 1000 times of that requirement. Finally, an additional limitation is that the expiration time and PEEP cannot be controlled independently. While we have explored with idea of including an additional needle valve on the expiration circuit to independently control these variables, doing so added significant relative resistance due to the much lower expiratory pressure gradient compared to that of the inspiratory circuit (20 mmHg vs. >1000 mmHg). Consequently, if we were to add this additional needle valve, we could compensate for the extra resistance by increasing the pressure gradient of the expiratory circuit via a vacuum on the output of the circuit. However, in our estimation, we deemed this to add excessive complexity and potential danger, as exposing the lung to a large amount of negative pressure could yield devastating and potentially lethal results. Therefore, for our design intended for emergency use, we opted to not include this additional feature on the expiratory circuit.

It is also important to note that, with regards to the biosafety of the industrial components used, our prototype system is still far from comparable to medically approved and clinically proven commercial ventilators. The purpose of the ALIVE Vent was to present an openly available design for use in emergency situations, yet many potential issues remain with regards to using such a system in its presented embodiment in humans, particularly concerning the use of non-medical grade components. A number of potential issues that could result in medium to long-term failure include corrosion, microbial growth, and human exposure to non-biocompatible substances.

Nonetheless, large animal testing is a crucial step toward clinical translation, and future work will implement safety features in both the software and hardware subsystems to fulfill the final requirements for hospital use. To realize widespread implementation of the proposed device, a deep, humanitarian-driven collaboration will be needed with industry partners that have the resources and expertise to translate our design into rapidly scalable assemblies, reduce costs, and implement mass production. While achieving these goals requires significant collaboration and industrial commitment, we remain optimistic in that our foundational research has provided the design inspiration and preliminary results to make this device a reality.

## Conclusions

As the global COVID-19 pandemic continues to spread, clinically viable, cost-effective, and rapidly scalable ventilator solutions must be implemented to avoid massive loss of life. Particularly in resource-scarce regions with limited access to mechanical ventilators, we envision the ALIVE Vent to help alleviate these shortages, with this solution now much closer to clinical translation due to our large animal testing results. Since we prioritize the accessibility of this design for massive global implementation, we have ensured that all components are publicly available. We strongly believe that while this pandemic has illuminated the enormous inequalities of healthcare systems, innovative, cost-effective solutions aimed at reducing socio-economic barriers, such as the ALIVE Vent, can help alter the narrative of inequality and enable access to prompt healthcare and life saving measures on a global scale and even beyond COVID-19.

## Supplementary Information

Below is the link to the electronic supplementary material.

Supplementary file 1 (DOCX 320 KB)

## Data Availability

All data of this paper are contained in the manuscript. Additionally, further information regarding technical details for openly available reproduction of this work (i.e., code, hardware designs, etc.) can be found at the online repository shared below. https://github.com/woo-laboratory-stanford/ALIVEVent.
