# Supplementary material for: From hardware store to hospital: a COVID-19-inspired, cost-effective, open-source, in vivo-validated ventilator for use in resource-scarce regions
Source: Biodes Manuf. 2021 Sep 22;5(1):133–40. doi: 10.1007/s42242-021-00164-1 (PMC8455802; doi:10.1007/s42242-021-00164-1)
Supplement: Supplementary file 1 — (DOCX 320 KB) [file 42242_2021_164_MOESM1_ESM.docx]

**SUPPLEMENTARY MATERIALS**

**
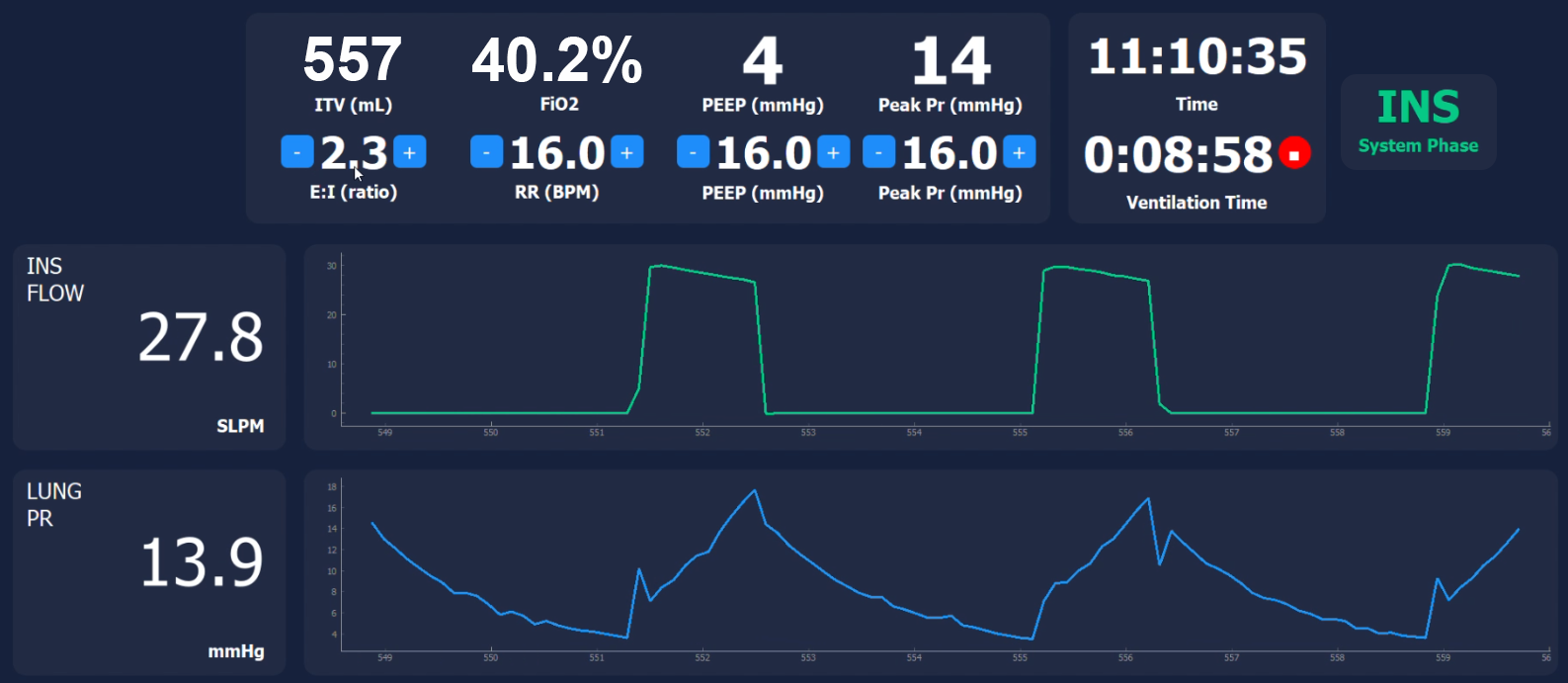
Fig. S1.** **Software Graphical Interface**. The custom software graphical interface designed for the ALIVE Vent system. The software was written in Python and the user interface uses the PyQt5 library.

**Table S1. AARC SARS CoV-2 Guidance of Ventilator Standard Functional Performance Requirements.** Standard performance requirements for ventilators stockpiled for use in mass casualty respiratory failure provided by the AARC SARS CoV-2 guidance document. (https://www.aarc.org/wp-content/uploads/2020/03/guidance-document-SARS-COVID19.pdf)

| **Operating Settings** | **Minimum** | **Maximum** |
| --- | --- | --- |
| Vt (mL) (4-8 mL/kg) | 50 | 750 |
| PEEP (mmHg) | 0 | 15 |
| Breathing Frequency (bpm) | 6 | 35 |
| FiO2 | 0.21 | 0.95 |
| Inspiratory Flow (LPM) | 10 | 80 |

**Table S2. Functional ALIVE Vent Testing Results with SmartLung.** The SmartLung consists of four independently adjustable levels of resistance (5, 20, 50, and 200 mbar/L/s) and compliance (10, 15, 20, and 30 mL/mbar at 400 mL V_T_). Given the maximum and minimum tidal volume (V_T_) (50, 750 mL) and respiratory rate (RR) levels (6, 20, and 35 bpm) set by the AARC guidance document for mass casualty respiratory failure ventilator stockpiling, we measured the maximum and average inspiratory flows (IF – Max and IF – Avg) for each of the 96 combinations of these four independent variables. Units for each column are as follows: Compliance (mL/mbar at 400 mL V_T_), Resistance (mbar/L/s), RR (bpm), V_T_ (mL), IF – Max (LPM), IF – Avg (LPM).

| **Compliance** | **Resistance** | **RR** | **V_T_** | **IF - Max** | **IF - Avg** |
| --- | --- | --- | --- | --- | --- |
| 30 | 5 | 6 | 46.0 | 1.9 | 1.0 |
| 30 | 20 | 6 | 49.0 | 1.7 | 0.9 |
| 30 | 50 | 6 | 45.0 | 1.6 | 0.8 |
| 30 | 200 | 6 | 45.0 | 1.6 | 0.8 |
| 30 | 5 | 6 | 751.0 | 15.8 | 13.5 |
| 30 | 20 | 6 | 750.0 | 15.7 | 13.5 |
| 30 | 50 | 6 | 754.0 | 15.5 | 13.6 |
| 30 | 200 | 6 | 759.0 | 16.8 | 13.7 |
| 20 | 5 | 6 | 45.0 | 1.6 | 0.8 |
| 20 | 20 | 6 | 45.0 | 1.7 | 0.8 |
| 20 | 50 | 6 | 44.0 | 1.7 | 0.8 |
| 20 | 200 | 6 | 44.0 | 1.6 | 0.8 |
| 20 | 5 | 6 | 752.3 | 16.7 | 13.5 |
| 20 | 20 | 6 | 754.7 | 16.7 | 13.6 |
| 20 | 50 | 6 | 752.1 | 16.0 | 13.5 |
| 20 | 200 | 6 | 752.8 | 16.8 | 13.6 |
| 15 | 5 | 6 | 45.8 | 1.6 | 0.8 |
| 15 | 20 | 6 | 45.1 | 1.7 | 0.8 |
| 15 | 50 | 6 | 45.4 | 1.7 | 0.8 |
| 15 | 200 | 6 | 44.9 | 1.7 | 0.8 |
| 15 | 5 | 6 | 754.1 | 17.6 | 13.6 |
| 15 | 20 | 6 | 750.0 | 17.4 | 13.5 |
| 15 | 50 | 6 | 753.1 | 16.9 | 13.6 |
| 15 | 200 | 6 | 756.0 | 17.1 | 13.6 |
| 10 | 5 | 6 | 48.3 | 1.8 | 0.9 |
| 10 | 20 | 6 | 47.1 | 1.8 | 0.8 |
| 10 | 50 | 6 | 45.8 | 1.8 | 0.8 |
| 10 | 200 | 6 | 45.6 | 1.7 | 0.8 |
| 10 | 5 | 6 | 755.4 | 19.2 | 13.6 |
| 10 | 20 | 6 | 754.1 | 19.1 | 13.6 |
| 10 | 50 | 6 | 752.6 | 18.5 | 13.5 |
| 10 | 200 | 6 | 751.8 | 17.7 | 13.5 |
| 30 | 5 | 20 | 45.0 | 2.9 | 2.7 |
| 30 | 20 | 20 | 44.0 | 2.9 | 2.6 |
| 30 | 50 | 20 | 48.0 | 3.0 | 2.8 |
| 30 | 200 | 20 | 47.0 | 3.2 | 2.8 |
| 30 | 5 | 20 | 750.0 | 46.9 | 45.0 |
| 30 | 20 | 20 | 754.0 | 47.8 | 45.2 |
| 30 | 50 | 20 | 750.0 | 47.9 | 45.0 |
| 30 | 200 | 20 | 751.0 | 55.7 | 45.9 |
| 20 | 5 | 20 | 48.0 | 3.1 | 2.9 |
| 20 | 20 | 20 | 48.0 | 3.2 | 2.9 |
| 20 | 50 | 20 | 46.0 | 3.1 | 2.7 |
| 20 | 200 | 20 | 50.0 | 3.2 | 3.0 |
| 20 | 5 | 20 | 754.8 | 47.4 | 45.3 |
| 20 | 20 | 20 | 754.0 | 47.4 | 45.2 |
| 20 | 50 | 20 | 753.0 | 48.5 | 45.2 |
| 20 | 200 | 20 | 752.8 | 54.8 | 45.2 |
| 15 | 5 | 20 | 48.3 | 3.5 | 2.9 |
| 15 | 20 | 20 | 48.0 | 3.3 | 2.9 |
| 15 | 50 | 20 | 47.2 | 3.5 | 2.8 |
| 15 | 200 | 20 | 45.0 | 3.2 | 2.7 |
| 15 | 5 | 20 | 756.5 | 47.9 | 45.4 |
| 15 | 20 | 20 | 751.8 | 47.8 | 45.1 |
| 15 | 50 | 20 | 757.2 | 49.4 | 45.4 |
| 15 | 200 | 20 | 758.5 | 55.2 | 45.5 |
| 10 | 5 | 20 | 47.7 | 3.3 | 2.9 |
| 10 | 20 | 20 | 47.0 | 3.3 | 2.8 |
| 10 | 50 | 20 | 47.7 | 3.4 | 2.9 |
| 10 | 200 | 20 | 49.7 | 3.4 | 3.0 |
| 10 | 5 | 20 | 754.2 | 49.5 | 45.2 |
| 10 | 20 | 20 | 752.0 | 48.9 | 45.1 |
| 10 | 50 | 20 | 751.7 | 48.8 | 45.1 |
| 10 | 200 | 20 | 754.8 | 55.1 | 45.3 |
| 30 | 5 | 35 | 49.0 | 5.6 | 5.1 |
| 30 | 20 | 35 | 48.0 | 5.6 | 5.3 |
| 30 | 50 | 35 | 49.0 | 5.5 | 5.0 |
| 30 | 200 | 35 | 48.0 | 5.4 | 5.1 |
| 30 | 5 | 35 | 756.0 | 87.3 | 79.4 |
| 30 | 20 | 35 | 769.0 | 90.2 | 80.8 |
| 30 | 50 | 35 | 752.0 | 86.6 | 78.9 |
| 30 | 200 | 35 | 758.0 | 101.1 | 79.6 |
| 20 | 5 | 35 | 50.0 | 5.7 | 5.2 |
| 20 | 20 | 35 | 50.0 | 5.7 | 5.2 |
| 20 | 50 | 35 | 49.0 | 5.5 | 5.2 |
| 20 | 200 | 35 | 50.0 | 5.6 | 5.2 |
| 20 | 5 | 35 | 758.0 | 83.9 | 79.6 |
| 20 | 20 | 35 | 758.7 | 84.1 | 79.7 |
| 20 | 50 | 35 | 755.7 | 92.0 | 79.3 |
| 20 | 200 | 35 | 752.4 | 100.8 | 79.0 |
| 15 | 5 | 35 | 48.3 | 5.6 | 5.1 |
| 15 | 20 | 35 | 48.7 | 5.7 | 5.1 |
| 15 | 50 | 35 | 47.3 | 5.5 | 5.0 |
| 15 | 200 | 35 | 46.2 | 5.3 | 4.8 |
| 15 | 5 | 35 | 757.5 | 82.9 | 79.5 |
| 15 | 20 | 35 | 751.9 | 83.5 | 79.0 |
| 15 | 50 | 35 | 759.5 | 84.7 | 79.8 |
| 15 | 200 | 35 | 753.0 | 99.5 | 79.1 |
| 10 | 5 | 35 | 48.6 | 5.6 | 5.1 |
| 10 | 20 | 35 | 47.3 | 5.5 | 5.0 |
| 10 | 50 | 35 | 47.3 | 5.5 | 5.0 |
| 10 | 200 | 35 | 47.8 | 5.5 | 5.0 |
| 10 | 5 | 35 | 756.5 | 82.1 | 79.4 |
| 10 | 20 | 35 | 758.4 | 81.9 | 79.6 |
| 10 | 50 | 35 | 756.8 | 86.3 | 79.5 |
| 10 | 200 | 35 | 753.3 | 99.8 | 79.1 |

**Table S3. In Vivo Test Results.** Arterial blood gas results from the in vivo testing. Time points 1, 2, and 3 roughly correspond to 20, 40, and 60 minutes after the start of ventilation.

| **ABG** | | | | | |  |
| --- | --- | --- | --- | --- | --- | --- |
|  | pH | pCO2 (mmHg) | pO2 (mmHg) | HCO3 -act (mmol/L) | BE(B) (mmol/L) | FiO_2_ |
| **Sheep 1 (40 kg)** |  |  |  |  |  |  |
| Baseline (Standard Vent) | 7.484 | 33.1 | 236 | 24.6 | 1.4 | 100% |
| **ALIVE Vent: Time point 1** | **7.432** | **35.8** | **147** | **23.4** | **-0.3** | **40%** |
| **ALIVE Vent: Time point 2** | **7.415** | **35.5** | **149** | **22.3** | **-1.6** |  |
| **ALIVE Vent: Time point 3** | **7.398** | **38.2** | **164** | **23** | **-1.1** |  |
| **Standard Vent: Time point 1** | **7.45** | **34.3** | **241** | **23.5** | **0** | **100%** |
| **Standard Vent: Time point 2** | **7.458** | **33.2** | **237** | **23.2** | **-0.2** |  |
| **Standard Vent: Time point 3** | **7.43** | **34.9** | **237** | **22.8** | **-0.9** |  |

| **ABG** | | | | | |  |
| --- | --- | --- | --- | --- | --- | --- |
|  | pH | pCO2 (mmHg) | pO2 (mmHg) | HCO3 -act (mmol/L) | BE(B) (mmol/L) | FiO_2_ |
| **Sheep 2 (60 kg)** |  |  |  |  |  |  |
| Baseline (Standard Vent) | 7.447 | 38.8 | 406.4 | 26.2 | 2 | 100% |
| **ALIVE Vent: Time point 1** | **7.45** | **41.6** | **120.7** | **28.3** | **3.9** | **40%** |
| **ALIVE Vent: Time point 2** | **7.444** | **43.2** | **127.2** | **28.9** | **4.4** |  |
| **ALIVE Vent: Time point 3** | **7.444** | **38.3** | **130.1** | **25.7** | **1.5** |  |
| **Standard Vent: Time point 1** | **7.449** | **40** | **481.6** | **27.1** | **2.9** | **100%** |
| **Standard Vent: Time point 2** | **7.453** | **43.3** | **490.6** | **29.6** | **5.2** |  |
| **Standard Vent: Time point 3** | **7.439** | **41** | **497** | **27.2** | **2.8** |  |

**Fig. S2. ALIVE Vent Bill of Materials (BOM)** (**A­­**) BOM and cost breakdown of the ALIVE Vent used in our testing setup. Note that electronics, microcontrollers, and computing equipment were not included in the final BOM because implementation will vary largely based on the final manifestation and the cost is minimal compared to the part cost. For example, computing could be executed using a $100 Raspberry Pi or a smartphone. (**B**) BOM and cost breakdown of the proposed ALIVE Vent to optimize for cost-efficiency. Most of the reduction in cost is due to the use of a medical-grade electro-galvanic oxygen sensor as opposed to a zirconia oxygen sensor, which we chose to use as to not contribute to the oxygen transducer shortage. Note that electronics, microcontrollers, and computing equipment were not included in the final BOM because implementation will vary largely based on the final manifestation. For example, computing could be implemented using a $100 Raspberry Pi or a smartphone.

**A**

| **Item** | **Quantity** | **Estimated Price** | **Supplier** | **Hyperlink** |
| --- | --- | --- | --- | --- |
| Push-to-Connect Needle Valve | 2 | $ 72.00 | McMaster-Carr | [Link](https://www.mcmaster.com/3019N114/) |
| Push-to-Connect Straight NPT Adapter | 8 | $ 40.00 | McMaster-Carr | [Link](https://www.mcmaster.com/5779K116/) |
| Push-to-Connect Straight BSPT Adapter | 1 | $ 15.00 | McMaster-Carr | [Link](https://www.mcmaster.com/5779K392/) |
| Push-to-Connect Tee Adapter | 2 | $ 14.00 | McMaster-Carr | [Link](https://www.mcmaster.com/5779K36/) |
| Push-to-Connect Panel Mount Adapter | 5 | $ 65.00 | McMaster-Carr | [Link](https://www.mcmaster.com/5779K273/) |
| Push-to-Connect Elbow to BSPT Adapter | 1 | $ 10.00 | McMaster-Carr | [Link](https://www.mcmaster.com/5779K432/) |
| Push-to-Connect Elbow Adapter | 3 | $ 18.00 | McMaster-Carr | [Link](https://www.mcmaster.com/5779K26/) |
| Push-to-Connect Elbow to NPT Adapter | 1 | $ 8.00 | McMaster-Carr | [Link](https://www.mcmaster.com/5779K638/) |
| Firm Polyurethane Tubing | 0.1 | $ 2.60 | McMaster-Carr | [Link](https://www.mcmaster.com/5648K26-5648K261/) |
| Stainless Steel NPT Union | 3 | $ 39.00 | McMaster-Carr | [Link](https://www.mcmaster.com/4513K91-4513K91) |
| 2-Way Solenoid Valve | 2 | $ 164.00 | McMaster-Carr | [Link](https://www.mcmaster.com/4738K151/) |
| Pressure Regulator | 2 | $ 188.00 | McMaster-Carr | [Link](https://www.mcmaster.com/1888k1-1888K11) |
| Flow Sensor | 1 | $ 137.00 | Honeywell | [Link](https://www.onlinecomponents.com/honeywell/hafuht0100l4axt-46961928.html) |
| Pressure Sensor | 1 | $ 200.00 | Cynergy3 | [Link](https://www.onlinecomponents.com/cynergy3/ipslugp001d-48159695.html) |
| Zirconia O2 Sensor | 1 | $ 200.00 | CO2 Meter | [Link](https://www.co2meter.com/products/zirconia-oxygen-sensor-system) |
| Zirconia O2 Sensor – Interface Board | 1 | $ 200.00 | CO2 Meter | [Link](https://www.co2meter.com/products/zirconia-oxygen-sensor-system?variant=31599941451894) |
| **Total** |  | **$ 1,372.60** |  |  |

**B**

| **Item** | **Quantity** | **Estimated Price** | **Supplier** | **Hyperlink** |
| --- | --- | --- | --- | --- |
| Push-to-Connect Straight NPT Adapter | 8 | $ 40.00 | McMaster-Carr | [Link](https://www.mcmaster.com/5779K116/) |
| Push-to-Connect Straight BSPT Adapter | 1 | $ 15.00 | McMaster-Carr | [Link](https://www.mcmaster.com/5779K392/) |
| Push-to-Connect Tee Adapter | 2 | $ 14.00 | McMaster-Carr | [Link](https://www.mcmaster.com/5779K36/) |
| Push-to-Connect Panel Mount Adapter | 5 | $ 65.00 | McMaster-Carr | [Link](https://www.mcmaster.com/5779K273/) |
| Push-to-Connect Elbow to BSPT Adapter | 1 | $ 10.00 | McMaster-Carr | [Link](https://www.mcmaster.com/5779K432/) |
| Push-to-Connect Elbow Adapter | 3 | $ 18.00 | McMaster-Carr | [Link](https://www.mcmaster.com/5779K26/) |
| Push-to-Connect Elbow to NPT Adapter | 1 | $ 8.00 | McMaster-Carr | [Link](https://www.mcmaster.com/5779K638/) |
| Firm Polyurethane Tubing | 0.1 | $ 2.60 | McMaster-Carr | [Link](https://www.mcmaster.com/5648K26-5648K261/) |
| Stainless Steel NPT Union | 3 | $ 39.00 | McMaster-Carr | [Link](https://www.mcmaster.com/4513K91-4513K91) |
| 2-Way Solenoid Valve | 2 | $ 164.00 | McMaster-Carr | [Link](https://www.mcmaster.com/4738K151/) |
| Pressure Regulator | 2 | $ 188.00 | McMaster-Carr | [Link](https://www.mcmaster.com/1888k1-1888K11) |
| Pressure Sensor | 1 | $ 200.00 | Cynergy3 | [Link](https://www.onlinecomponents.com/cynergy3/ipslugp001d-48159695.html) |
| Flow Sensor | 1 | $ 137.00 | Honeywell | [Link](https://www.onlinecomponents.com/honeywell/hafuht0100l4axt-46961928.html) |
| Electro-galvanic Oxygen Sensor | 1 | $ 76.00 | Puritan Bennett | [Link](https://www.cablesandsensors.com/products/compatible-o2-cell-for-hamilton-medical-396008?variant=33810165512) |
| **Total** |  | **$ 976.60** |  |  |
